# Adipose Tissue Insulin Resistance: A Key Driver of Metabolic Syndrome Pathogenesis

**DOI:** 10.3390/biomedicines13102376

**Published:** 2025-09-28

**Authors:** Atefeh Rabiee, Md Arafat Hossain, Ankita Poojari

**Affiliations:** Department of Pharmaceutical Sciences, Thomas J. Long School of Pharmacy, University of the Pacific, Stockton, CA 95211, USA

**Keywords:** adipose tissue insulin resistance, metabolic syndrome, adipokine signaling, inflammation, obesity, type 2 diabetes, insulin sensitivity, adipocyte dysfunction, metabolic dysfunction, therapeutic targets

## Abstract

Metabolic syndrome (MetS), characterized by obesity, insulin resistance, dyslipidemia, and hypertension, is a growing global health concern. This review examines the relationship between adipose tissue insulin resistance (AT-IR) and MetS. Adipose tissue functions beyond energy storage as an endocrine organ that regulates metabolism through hormone and cytokine secretion. When adipose tissue becomes insulin resistant, it contributes to systemic metabolic dysfunction through impaired glucose uptake and dysregulated adipokine production. This creates a bidirectional relationship where AT-IR promotes MetS development, while MetS-associated inflammation further worsens adipose insulin sensitivity. Key mechanisms include inflammatory signaling, altered adipokine profile, and mitochondrial dysfunction. Understanding these interactions offers therapeutic opportunities, as targeting adipose tissue function may provide novel approaches for MetS treatment. This review synthesizes current evidence on AT-IR-MetS interactions and discusses therapeutic implications and future research directions.

## 1. Introduction

Metabolic syndrome (MetS) impacts ~30% of adults worldwide. It poses a significant public health challenge [1]. This condition clusters cardiovascular risk factors. It includes obesity, hypertension, dyslipidemia, and insulin resistance. Research highlights AT-IR as exhibiting a fundamental regulatory function, redefining adipose tissue as dynamic endocrine modulator that regulates systemic metabolism [2].

AT-IR initiates a cascade of metabolic dysfunction, contributing to the development of MetS [3]. Unlike muscle or liver insulin resistance, AT-IR emerges later. It can be detected early, offering an intervention window [4,5]. Early AT-IR targeting may prevent progression. It addresses severe conditions like type 2 diabetes and cardiovascular disease [6].

Adipose tissue varies by location, with distinct metabolic roles. Visceral fat, surrounding internal organs, is more inflammatory and linked to greater metabolic risk than subcutaneous fat [7,8,9,10]. These differences explain variations in metabolic outcomes among individuals with similar body weight [11]. AT-IR and MetS form a feedback loop, where impaired fat storage leads to ectopic lipid accumulation in other tissues, such as liver and muscle, exacerbating systemic insulin resistance [12,13,14,15]. This process accelerates MetS progression [16].

Advanced technologies, including multi-omics and imaging, have clarified the molecular and inflammatory pathways linking AT-IR to MetS [17,18,19]. These insights guide the development of targeted therapies, supporting personalized approaches to manage metabolic disorders [20]. This review aligns with *Biomedicines’* mission to advance translational research by exploring AT-IR mechanisms and therapeutic innovations for metabolic disorders.

## 2. Historical Perspective and Evolution of Concepts

Adipose tissue was recognized as an endocrine organ. This began with leptin’s discovery by Zhang et al. in 1994 [21]. This discovery marked a foundational paradigm shift in understanding adipose biology. Hotamisligil and colleagues (1993) demonstrated TNF-α production by adipose tissue [22]. This established adipose inflammation’s role in insulin resistance.

**Figure 1:**
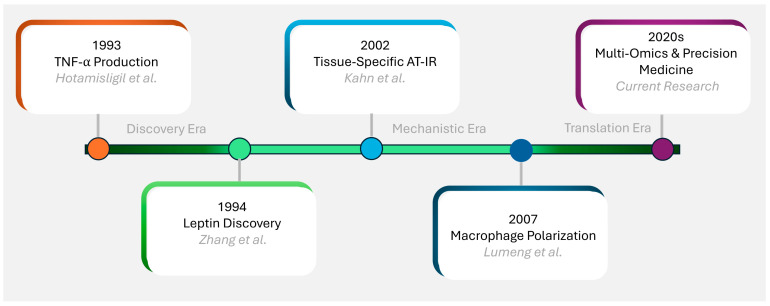
Historical timeline of key discoveries in adipose tissue biology: Timeline showing major discoveries in adipose tissue biology and insulin resistance research. Key milestones include identification of TNF-α production by adipose tissue (1993) [22], the discovery of leptin (1994) [21], the establishment of tissue-specific insulin resistance concepts (2002) [24] and macrophage depolarization [25]. Recent advances include multi-omics understanding of adipose tissue heterogeneity and therapeutic targets. Color coding represents different research eras: orange (inflammatory discovery), green (endocrine function), light blue (mechanistic understanding), dark blue (immune interactions), and purple (translational applications).

**Figure 2:**
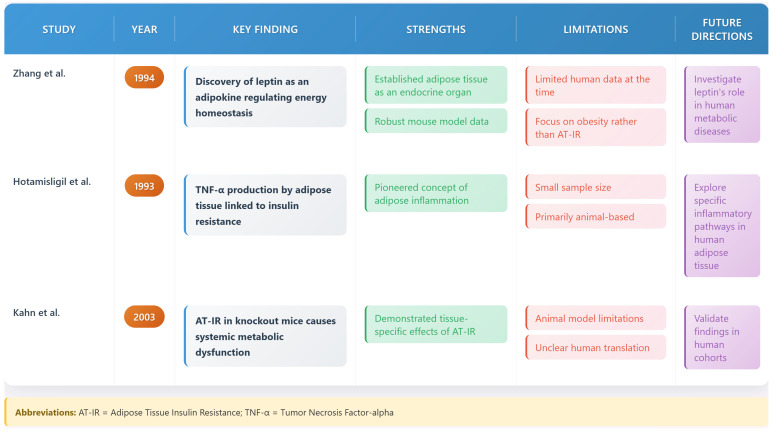
Key studies in adipose tissue biology: The figure highlights pivotal studies (Zhang et al., 1994 [21]; Hotamisligil et al., 1993 [22]; Kahn et al., 2003 [23]) that shaped understanding of adipose tissue as an endocrine organ and its role in insulin resistance. It includes key findings, strengths, limitations, and future research directions.

The specific concept of AT-IR emerged from the work of Kahn and colleagues (2002–2003) [23,24], who demonstrated that tissue-specific insulin receptor-knockout mice developed distinct metabolic phenotypes. Their research established that AT-IR alone could precipitate systemic metabolic dysfunction, even when insulin signaling remained intact in other tissues. The timeline of these major discoveries illustrates the evolution of our understanding of adipose tissue biology and its central role in metabolic disease [21,22,23,24] (see Figure 1 and Figure 2).

## 3. Molecular Mechanisms of Adipose Tissue Insulin Resistance

### 3.1. Insulin Signaling in Healthy Adipose Tissue

In healthy adipose tissue, insulin signaling regulates glucose uptake and lipid storage. It controls gene expression via IRS proteins, PI3K, and Akt [26]. This pathway promotes glucose transporter movement to the cell surface, inhibits fat breakdown via hormone-sensitive lipase, and controls metabolic gene activity.

Recent studies highlight the precision of this process. Insulin receptors cluster in specific membrane regions to optimize signaling efficiency, as shown by advanced imaging techniques [27]. These findings underscore the spatial organization of insulin signaling in adipocytes, critical for maintaining metabolic balance.

### 3.2. Disruption of Insulin Signaling in AT-IR

In adipose tissue insulin resistance (AT-IR), inflammation exerts a dominant disruptive influence on insulin signaling, impairing glucose uptake and lipid storage. Pro-inflammatory molecules, such as cytokines, activate enzymes like JNK and IKK-β, which alter insulin receptor substrate (IRS) proteins, reducing their ability to transmit insulin signals [28]. This disruption prevents effective glucose transport and increases fat breakdown, contributing to metabolic dysfunction.

**Figure 3:**
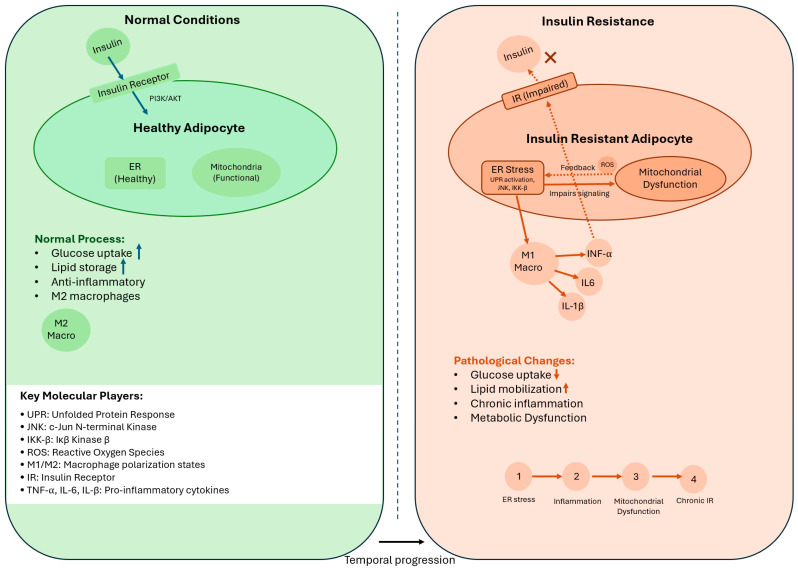
Molecular mechanisms of adipose tissue insulin resistance: Schematic illustrating the interplay of ER stress, mitochondrial dysfunction, and inflammation in AT-IR. Under normal conditions (**left**), insulin signaling promotes glucose uptake and lipid storage. In insulin resistance (**right**), ER stress initiates the cascade by activating UPR and stress kinases (JNK, IKK-β), leading to inflammation via M1 macrophage recruitment and cytokine production. Mitochondrial dysfunction, driven by ROS, sustains chronic impairment, forming a feedback loop with ER stress. Solid arrows indicate direct molecular signaling pathways and temporal progression, while dashed arrows represent feedback mechanisms. “X” on the right panel indicates the condition when the connection between insulin and its’ receptors is impaired. In both panels, arrows pointing up indicate the upregulation and arrows pointing down indicate the downregulation.

Studies show that specific immune cells, called macrophages, play a key role in this process. Research by Lumeng and colleagues in 2007 identified two types of macrophages in adipose tissue: M1 macrophages, which produce harmful cytokines and reactive oxygen species (ROS) that worsen insulin resistance, and M2 macrophages, which may support healthier tissue function [25]. Figure 3 illustrates these pathways, showing how inflammation, endoplasmic reticulum stress, and mitochondrial dysfunction converge to impair adipose tissue’s metabolic role [25,28]. These findings highlight inflammation as a central driver of AT-IR, linking it to broader metabolic disorders.

### 3.3. Cellular Stress Pathways

#### 3.3.1. Endoplasmic Reticulum Stress

ER stress affects adipose tissue dysfunction. Özcan et al. (2004) showed that obesity induces ER stress, triggering UPR and ISR pathways [29]. These pathways disrupt insulin signaling and promote inflammation. Recent studies by Zhou and colleagues (2009) further demonstrate that ER stress in adipocytes activates complex cellular responses, exacerbating AT-IR [30]. Chronic ER stress promotes polarization of adipose tissue-resident macrophages to a pro-inflammatory M1 phenotype, exacerbating adipocyte insulin resistance [31].

#### 3.3.2. Mitochondrial Dysfunction

Mitochondrial dysfunction represents another key aspect of AT-IR. Research by Anderson et al. (2009) demonstrated that excessive nutrient intake leads to mitochondrial stress and increased production of reactive oxygen species (ROS) [32]. These changes impair insulin signaling through multiple mechanisms, including direct oxidative damage to signaling proteins and activation of stress-responsive kinases. A reduced abundance of mitochondria and expression of mitochondrial genes have been reported in patients with insulin resistance. White adipose tissue (WAT) of these patients was observed to have decreased oxygen consumption and ATP production [33,34].

### 3.4. Integration of Stress Pathways in AT-IR

The development of adipose tissue insulin resistance (AT-IR) involves a complex interplay of endoplasmic reticulum (ER) stress, mitochondrial dysfunction, and inflammation, which converge to disrupt insulin signaling. Temporally, ER stress often initiates the cascade, as nutrient overload triggers the unfolded protein response (UPR), leading to activation of pro-inflammatory pathways such as JNK and IKK-β [29]. This inflammatory response recruits M1 macrophages, which exacerbate tissue inflammation and produce reactive oxygen species (ROS), contributing to mitochondrial dysfunction [32]. Hierarchically, ER stress acts as an upstream trigger, while inflammation amplifies the response, and mitochondrial dysfunction sustains chronic metabolic impairment. Recent studies suggest that these pathways form a feedback loop, where mitochondrial ROS further exacerbate ER stress, creating a vicious cycle [30]. This integrated framework highlights the synergistic roles of these pathways in AT-IR and underscores the need for therapies targeting their convergence points, such as stress-responsive kinases or inflammatory mediators.

## 4. Adipokines and Metabolic Regulation

### 4.1. The Adipokine Network

Adipose tissue secretes numerous bioactive molecules collectively termed adipokines. A proteomic analysis by Lehr et al. (2012) [35] has identified over 600 proteins secreted by adipose tissue, many with previously unknown functions. These adipokines form a complex network that regulates systemic metabolism through endocrine, paracrine, and autocrine mechanisms. Adipokine dysregulation extends beyond a mere consequence of AT-IR, serving as a central diagnostic and prognostic tool. Visceral adipose tissue, linked to greater metabolic risk, exhibits a distinct ‘adipokine signature’—elevated pro-inflammatory cytokines TNF-α and IL-6, and reduced beneficial adiponectin compared to subcutaneous fat—detectable before overt metabolic disease [35]. A decline in circulating adiponectin alongside a rise in inflammatory markers provides early warning signs of metabolic risk, enhancing clinical monitoring and intervention strategies [35].

### 4.2. Key Adipokines in Metabolic Regulation

#### 4.2.1. Leptin

Leptin, the first discovered adipokine, plays a crucial role in energy homeostasis and metabolism. Recent work has revealed new aspects of leptin biology, including its role in regulating immune cell function and brown adipose tissue activation [36]. Leptin modulates hypothalamic signaling to control appetite and energy expenditure, and its dysregulation in obesity contributes to AT-IR [36]. Additionally, leptin influences adipose tissue remodeling by promoting angiogenesis and adipocyte differentiation, impacting systemic metabolic health [36].

#### 4.2.2. Adiponectin

Adiponectin, another key adipokine, has emerged as a critical regulator of insulin sensitivity. Studies by Holland and colleagues (2011) have identified novel mechanisms by which adiponectin improves insulin sensitivity, including effects on ceramide metabolism and mitochondrial function [37]. Adiponectin enhances glucose uptake in adipocytes and reduces hepatic gluconeogenesis, counteracting AT-IR [37]. Its anti-inflammatory properties also mitigate adipose tissue inflammation, supporting metabolic homeostasis [37].

**Figure 4:**
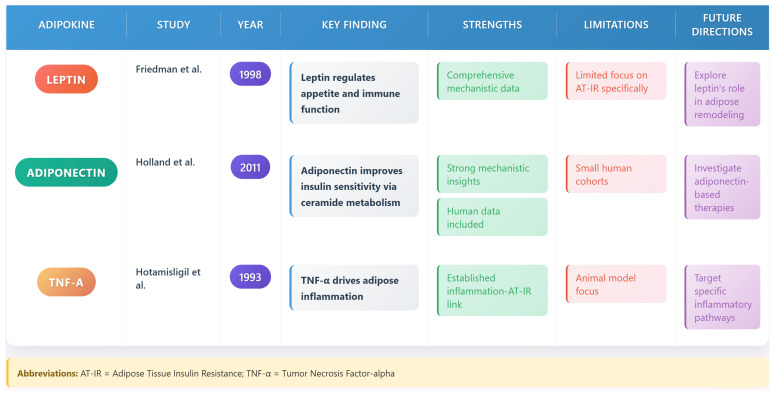
Key adipokine studies and their implications: The figure brings together key adipokine studies (Friedman et al., 1998 [38]; Holland et al., 2011 [37]; Hotamisligil et al., 1993 [22]) focusing on leptin, adiponectin, and TNF-α, outlining their findings, strengths, limitations, and future research directions in AT-IR and metabolic regulation.

Figure 4 summarizes the key adipokine studies and their implications.

## 5. Adipose Tissue Expansion and Remodeling

### 5.1. Healthy Versus Pathological Expansion

The manner in which adipose tissue expands in response to excess nutrients significantly influences metabolic health. Research by Beals et al. (2021) has demonstrated that healthy adipose tissue expansion involves coordinated increases in adipocyte size and number, accompanied by appropriate angiogenesis and extracellular matrix remodeling [39]. In contrast, pathological expansion is characterized by rapid adipocyte hypertrophy, insufficient vascularization, and excessive fibrosis. The adipose tissue expandability hypothesis suggests that adipocyte hypertrophy beyond a critical size induces hypoxia, triggering inflammation and impairing insulin signaling, a mechanical stress initiating the pathological cascade [39].

### 5.2. Extracellular Matrix Dynamics

The importance of extracellular matrix (ECM) remodeling in adipose tissue function has become increasingly apparent. Studies by Lin and colleagues (2016) have shown that ECM composition significantly influences adipocyfunction and insulin sensitivity [40]. Excessive collagen deposition, particularly of specific collagen subtypes, contributes to adipose tissue dysfunction and insulin resistance.

## 6. Disease States Associated with Adipose Tissue Insulin Resistance

Lipotoxicity emerges when adipose tissue fails to store excess lipids, spilling free fatty acids into organs like the liver, causing ectopic accumulation and insulin resistance, as seen in NAFLD’s hepatic steatosis [41]. This common denominator links AT-IR to diverse metabolic and non-metabolic diseases, where impaired lipid buffering exacerbates systemic dysfunction across T2DM, NAFLD, CVD, PCOS, and more.

### 6.1. Obesity and AT-IR

Obesity exacerbates AT-IR, contributing to metabolic dysfunction. As adipose tissue expands, it undergoes pathological changes that impair insulin signaling and increase inflammation [42]. Excessive lipid accumulation in adipocytes disrupts insulin sensitivity, while pro-inflammatory immune cells infiltrate the tissue, reducing adiponectin production and elevating inflammatory signals [42].

**Figure 5:**
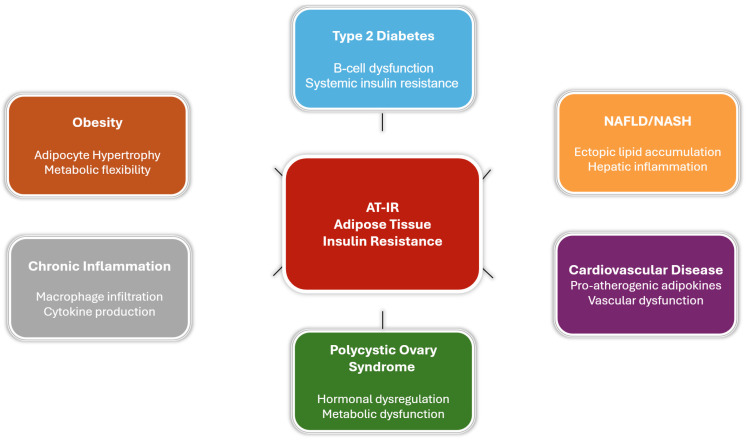
Conceptual diagram illustrates the central role of adipose tissue insulin resistance (AT-IR) in metabolic syndrome pathogenesis. AT-IR serves as the primary pathophysiological hub driving multiple disease states through distinct mechanisms. Each condition box includes the key mechanistic pathway by which AT-IR contributes to disease development. The central positioning emphasizes AT-IR as a unifying therapeutic target that could potentially address multiple components of metabolic syndrome simultaneously.

The location of fat accumulation significantly influences metabolic outcomes. Visceral obesity, characterized by fat around internal organs, is more inflammatory and insulin-resistant than subcutaneous obesity, increasing the risk of systemic metabolic disorders [43]. These differences highlight the global burden of MetS, with AT-IR playing a central role in its progression (see Figure 5) [1].

### 6.2. Type 2 Diabetes Mellitus

The progression from AT-IR to type 2 diabetes involves intricate bidirectional interactions between adipose tissue dysfunction and pancreatic β cell failure. Kim et al. (2023) have demonstrated that signals from insulin-resistant adipose tissue, including specific lipid species and inflammatory mediators, directly impair β cell function and insulin secretion [44].

Long-term studies by Semnani et al. (2021) have revealed that AT-IR often precedes the development of systemic insulin resistance and diabetes by several years, suggesting a potential window for therapeutic intervention [12]. Their work has identified several key markers of adipose tissue dysfunction that may serve as early warning signs for diabetes risk.

### 6.3. Non-Alcoholic Fatty Liver Disease (NAFLD)

The relationship between AT-IR and NAFLD has become increasingly clear through recent research. Studies by Lee et al. (2023) have shown that dysfunction in adipose tissue leads to increased lipolysis and flux of free fatty acids to the liver, promoting hepatic steatosis and inflammation [41]. Lipotoxicity, as detailed by Lee et al. (2023), links AT-IR to NAFLD progression through ectopic lipid accumulation [41]. Tordjman et al. showed that subcutaneous adipose tissue (SAT) of patients with NAFLD exhibits a specific signature with increased expression of inflammatory genes, ECM remodeling, and a decrease in adipogenic and oxidative metabolism genes [45].

The progression from simple steatosis to non-alcoholic steatohepatitis (NASH) appears to be influenced by adipose tissue-derived factors. Work by Micu and colleagues (2021) has identified specific adipokine patterns associated with NASH progression, suggesting potential therapeutic targets [46].

However, the strength of evidence varies, with human studies often limited by small sample sizes and reliance on associative data, while animal models may overestimate the direct role of AT-IR due to species-specific metabolic differences. For instance, discrepancies exist regarding the relative contributions of visceral versus subcutaneous fat to NAFLD progression, with some studies suggesting visceral fat as the primary driver [47], while others highlight subcutaneous fat’s role in systemic inflammation [48]. Key knowledge gaps include the precise adipokine profiles driving NAFLD and the lack of longitudinal human studies to confirm causality. These limitations underscore the need for larger, well-controlled clinical studies to validate preclinical findings and clarify therapeutic targets.

### 6.4. Cardiovascular Disease

AT-IR plays a crucial role in the development of cardiovascular disease through multiple mechanisms. Research by Chait et al. (2020) has demonstrated that insulin-resistant adipose tissue produces a pro-atherogenic profile of inflammatory mediators and adipokines [8].

Recent studies have particularly focused on the role of perivascular adipose tissue. Work by Adachi et al. (2023) has shown that insulin resistance in this specific adipose depot directly influences vascular function and atherosclerosis progression [49].

### 6.5. Polycystic Ovary Syndrome (PCOS)

The intertwined molecular pathways between AT-IR and PCOS are evident. Studies by Bril et al. (2024) have shown that adipose tissue dysfunction contributes to the hormonal and metabolic disturbances characteristic of PCOS [50]. Women with PCOS have abnormal adipocytes and perturbed adipocyte functionality, favoring insulin resistance and subclinical inflammation [51]. Their work suggests that targeting adipose tissue insulin sensitivity may represent a novel therapeutic approach for PCOS management.

However, conflicting evidence exists on whether AT-IR is a primary driver of PCOS or a secondary consequence of hyperandrogenism, with some studies suggesting bidirectional interactions [50]. The reliance on cross-sectional studies limits causal inferences, and small sample sizes in human trials hinder generalizability [50]. Moreover, controversies remain regarding the extent to which adipose tissue inflammation, as opposed to ovarian dysfunction, drives metabolic outcomes in PCOS [52]. Future research should focus on longitudinal studies and mechanistic investigations to resolve these inconsistencies and identify targeted interventions [52].

### 6.6. Cancer

AT-IR exerts a key oncogenic influence in cancer development. Research by Kim et al. (2024) has demonstrated that insulin-resistant adipose tissue creates a microenvironment that may promote tumor growth and progression [53]. The mechanisms involve altered adipokine production, increased inflammation, and changes in metabolic substrate availability [53].

**Figure 6:**
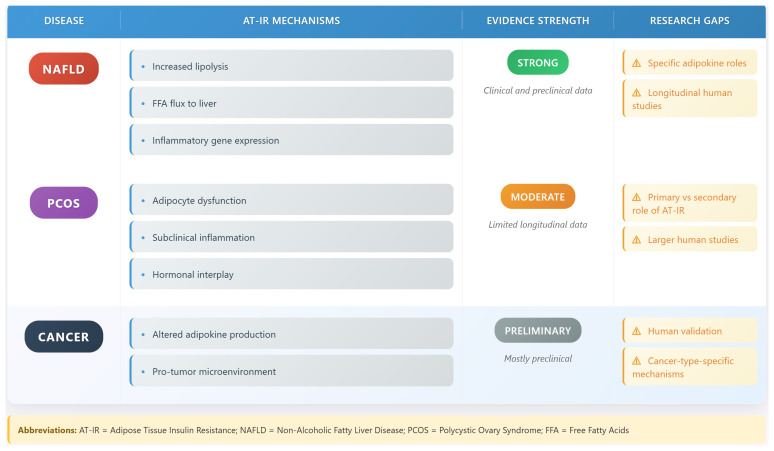
Comparative analysis of AT-IR in NAFLD, PCOS, and cancer. The figure compares AT-IR mechanisms in NAFLD (lipolysis, FFA flux, inflammation), PCOS (adipocyte dysfunction, inflammation), and cancer (altered adipokines, pro-tumor microenvironment), noting evidence strength and research gaps. The warning symbols (⚠) indicate research gaps—areas where more investigation is needed.

However, the evidence is predominantly derived from preclinical models, with limited human studies to confirm these associations [54]. Controversies exist regarding the specific adipokines (e.g., leptin versus adiponectin) driving cancer risk, as well as the role of AT-IR in different cancer types [53]. Methodological limitations, such as the lack of standardized measures for AT-IR in cancer patients, further complicate interpretation [54]. These gaps highlight the need for clinical studies to validate preclinical findings and explore cancer-type-specific mechanisms. Figure 6 compares AT-IR in NAFLD, PCOS, and cancer [54].

### 6.7. Age-Related Metabolic Dysfunction

AT-IR contributes significantly to age-related metabolic decline. Ou et al. (2022) have shown that aging is associated with specific changes in adipose tissue function and insulin sensitivity, including reduced adipocyte turnover and increased inflammatory signaling [55]. These alterations impair lipid storage and promote ectopic fat deposition, exacerbating metabolic dysfunction in older populations [55]. Targeting age-related AT-IR could mitigate the rising prevalence of metabolic disorders in aging societies.

### 6.8. Adipose Tissue Inflammation and Systemic Inflammatory Feedback in Insulin Resistance

Adipose tissue inflammation is a key driver of adipose tissue insulin resistance (AT-IR) and metabolic syndrome (MetS), exhibiting bidirectional relationships with chronic inflammatory diseases. Systemic inflammatory conditions, such as rheumatoid arthritis, amplify adipose tissue dysfunction by enhancing pro-inflammatory cytokine production (e.g., TNF-α and IL-6), which impairs insulin signaling [19,56]. Conversely, AT-IR exacerbates systemic inflammation, creating feedback loops that worsen metabolic and inflammatory outcomes [19,56]. This chronic low-grade immune activation is driven by immune cell infiltration in adipose tissue during obesity, particularly by M1-polarized macrophages, which release pro-inflammatory cytokines, while M2 macrophages promote anti-inflammatory effects and tissue homeostasis [41]. T-cells also play a role, with Th1 cells exacerbating inflammation and regulatory T-cells mitigating it [53]. Adipose tissue-resident dendritic cells and neutrophils further amplify this inflammatory milieu, with hypoxia-induced inflammation identified as a key mechanism in AT-IR progression [41] These findings highlight the therapeutic potential of targeting adipose tissue inflammation and immune modulation to improve outcomes for both metabolic and inflammatory disorders [37].

## 7. Metabolic Consequences of AT-IR

### 7.1. Systemic Effects

AT-IR has far-reaching effects on whole-body metabolism. Recent metabolomic studies by Mathioudaki et al. (2024) have revealed complex patterns of metabolite alterations associated with AT-IR, including changes in lipid species, amino acids, and various metabolic intermediates [57]. These alterations contribute to the development of systemic insulin resistance and metabolic dysfunction.

### 7.2. Tissue Crosstalk

The concept of inter-organ communication in metabolic regulation has gained increasing attention. Research by Piquet et al. (2022) has demonstrated that signals from insulin-resistant adipose tissue, including specific lipid species and inflammatory mediators, directly affect the function of other metabolic tissues such as liver, muscle, and pancreatic β-cells [58].

## 8. Therapeutic Approaches and Clinical Management

### 8.1. Current Therapeutic Strategies

#### 8.1.1. Lifestyle Interventions

Lifestyle interventions are essential for managing AT-IR. They include exercise and diet, enhancing insulin sensitivity and reducing inflammation. Regular physical activity enhances insulin sensitivity, improves glucose uptake, and reduces inflammation in adipose tissue [54]. Exercise also promotes mitochondrial function and transforms white fat into metabolically active brown-like fat, increasing energy expenditure [3].

These changes improve adipose tissue’s inflammatory profile by reducing pro-inflammatory signals and supporting beneficial immune cell activity. Additionally, exercise enhances blood vessel networks, ensuring efficient nutrients and oxygen delivery to adipocytes, which supports healthy tissue function [59].

#### 8.1.2. Pharmacological Interventions

Among established pharmacological approaches, thiazolidinediones (TZDs) represent well-characterized insulin sensitizers with specific adipose tissue effects. The therapeutic action of TZDs centers on peroxisome proliferator-activated receptor gamma (PPARγ) activation, which orchestrates beneficial changes in adipocyte biology [60]. This nuclear receptor activation promotes the differentiation of healthy, insulin-sensitive adipocytes while preventing excessive lipid accumulation in non-adipose tissues. However, TZDs are associated with side effects such as weight gain, fluid retention, and increased risk of heart failure, limiting their use in some patients. Additionally, their high cost and need for long-term monitoring pose accessibility challenges in resource-limited settings.

The therapeutic landscape has expanded significantly with the introduction of incretin-based medications. GLP-1 receptor agonists demonstrate multifaceted effects on adipose tissue biology, including anti-inflammatory actions and enhanced insulin sensitivity [61]. These agents also influence adipocyte metabolism by promoting beneficial adipokine production and supporting metabolic flexibility. The resulting improvements in adipokine secretion and reduced inflammatory signaling contribute to systemic metabolic benefits. GLP-1 receptor agonists, mimicking native GLP-1, trigger insulin release, block glucagon secretion, and slow stomach emptying, reducing blood glucose and caloric intake. This decreases metabolic load on adipose tissue, enabling dysfunctional adipocytes to recover, reducing inflammation, and improving insulin sensitivity [62,63]. Despite their efficacy, GLP-1 agonists can cause gastrointestinal side effects, and their high cost may restrict access for some populations.

Innovation in drug development has led to the creation of combination therapies that target multiple metabolic pathways simultaneously. Multi-receptor agonists combining GLP-1, GIP, and glucagon signaling represent a paradigm shift in metabolic disease treatment [64]. These agents demonstrate superior efficacy compared to single-target approaches, improving insulin sensitivity and reducing body weight. However, their side effects, including nausea and potential cardiovascular risks, require careful monitoring. The lack of long-term safety data, particularly beyond 2–3 years, remains a concern, and their high cost limits widespread adoption. Ongoing clinical trials are needed to establish their safety and cost-effectiveness for diverse patient populations.

### 8.2. Emerging Therapeutic Targets

Tissue-specific drug delivery systems target adipose tissue to improve therapeutic efficacy and minimize systemic side effects. Nanoparticle-based approaches enable selective delivery to adipose tissue, addressing its unique metabolic properties. RNA-based therapeutics, such as siRNA and antisense oligonucleotides, modulate specific pathways in adipose tissue with high precision. However, these emerging therapies face significant translational barriers, including high development costs, potential off-target effects, and challenges in achieving stable delivery to adipose tissue. For instance, siRNA therapies require sophisticated delivery systems to ensure specificity, and their long-term safety in humans remains under investigation. Regulatory hurdles and the need for large-scale clinical trials further delay their clinical implementation.

#### 8.2.1. Adipose Tissue Remodeling

Targeting extracellular matrix (ECM) composition is a promising strategy for addressing adipose tissue dysfunction. Modulating matrix components can restore healthy tissue architecture and improve insulin sensitivity [65]. Current research explores enzyme activation to promote beneficial matrix turnover and inhibition of excessive collagen buildup, creating a supportive environment for adipocyte function.

Enhancing vascular networks in adipose tissue is another key approach. Improved angiogenesis ensures adequate nutrient and oxygen delivery, supporting insulin-sensitive adipocytes [66]. These strategies aim to optimize adipose tissue health and mitigate metabolic dysfunction.

#### 8.2.2. Biomarker Development and Clinical Monitoring

Biomarker identification has improved the detection and monitoring of adipose tissue dysfunction. Metabolomic profiling identifies lipid signatures associated with insulin resistance, enabling early risk stratification and intervention [57]. Protein-based and microRNA biomarkers further enhance diagnostic capabilities for assessing adipose tissue health.

Advanced imaging techniques, such as positron emission tomography with metabolic tracers, measure tissue-specific insulin action and metabolic flux. Magnetic resonance imaging quantifies fat distribution and composition, providing detailed insights into adipose tissue function [57]. These tools, combined with molecular biomarkers, support comprehensive evaluation of adipose tissue health in clinical settings.

**Figure 7:**
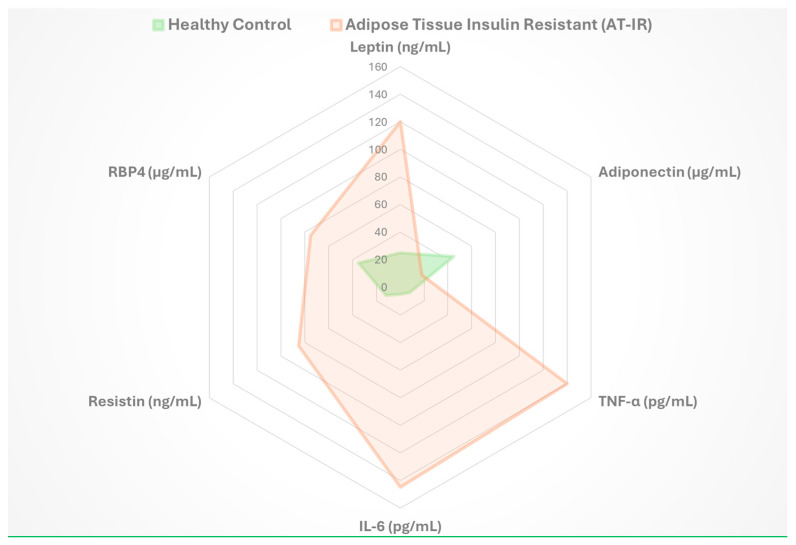
Biomarkers of adipose tissue insulin resistance. Radar plot comparing biomarker profiles between healthy controls and AT-IR subjects. Key biomarkers include adipokine (leptin [36], adiponectin [37]), inflammatory markers (TNF-α [22], IL-6 [46]), and metabolic indicators (Resistin, RBP4 [58]). The distinct AT-IR signature demonstrates adipose tissue-specific dysfunction patterns that differ from systemic insulin resistance.

Key biomarkers associated with AT-IR, including changes in adipokine levels and inflammatory markers, are summarized in Figure 7, which provides insights into adipose tissue dysfunction and metabolic health status.

#### 8.2.3. Integration of Multi-Omic Approaches

Multi-omic analyses, combining transcriptomics, proteomics, and metabolomics, reveal regulatory networks underlying AT-IR. These studies identify novel pathways and intervention points, enhancing our understanding of metabolic dysfunction [17].

Single-cell analysis highlights cellular diversity within adipose tissue, identifying distinct cell populations that shift during insulin resistance progression. Recent studies, such as Vamvini et al. (2024) [67], which employed spatial transcriptomics to demonstrate that exercise training and cold exposure induce distinct molecular adaptations in inguinal white adipose tissue, including proteome changes related to extracellular space and vesicle transport that correlate with improved glucose tolerance. Wang et al. (2025) [68] further integrated spatial transcriptomics with single-nucleus RNA-seq to reveal the spatial heterogeneity of intramuscular fat and the inhibitory role of TGF-β signaling in fat deposition under obese conditions, utilizing single-cell and spatial transcriptomics to map adipocyte heterogeneity and spatial interactions in AT-IR, revealing novel regulatory networks not captured in earlier studies like Massier et al. (2023) [69]. These findings clarify adipocyte precursor responses to metabolic stress, informing strategies for tissue remodeling and metabolic health restoration [69].

#### 8.2.4. Immune System Modulation in Adipose Tissue

The therapeutic targeting of immune dysfunction within adipose tissue has gained considerable attention as a strategy for metabolic disease treatment. Rather than employing broad immunosuppressive approaches, precision targeting specific inflammatory cascades offers greater therapeutic potential with reduced side effects [42]. This strategy recognizes that different immune cell populations within adipose tissue contribute uniquely to metabolic dysfunction, necessitating selective rather than comprehensive immune modulation.

Adipose tissue macrophages are a major contributor to inflammation in obesity. Targeting macrophage recruitment has shown promise in preliminary studies. For example, blocking CCL2/CCR2 has been shown to reduce macrophage recruitment and improve obesity-induced inflammation and insulin resistance [70]. Another approach involves switching polarization of macrophages from pro-inflammatory M1 to anti-inflammatory M2 by targeting known cytokine transcription factors [25]. An emerging therapeutic concept involves the enhancement of natural inflammation resolution mechanisms. Specialized lipid mediators that promote the active resolution of inflammatory processes represent a novel therapeutic class [71]. These molecules work by facilitating the clearance of inflammatory cells and promoting tissue repair, offering a fundamentally different approach compared to traditional anti-inflammatory strategies that simply suppress immune responses.

### 8.3. Clinical Translation and Implementation

The translation of basic research findings into clinical practice remains a critical challenge. Recent clinical trials have begun to evaluate novel therapeutic approaches based on our enhanced understanding of adipose tissue biology. These trials increasingly incorporate biomarker analyses and advanced imaging techniques to better understand treatment responses and identify patient subgroups most likely to benefit from specific interventions.

**Figure 8:**
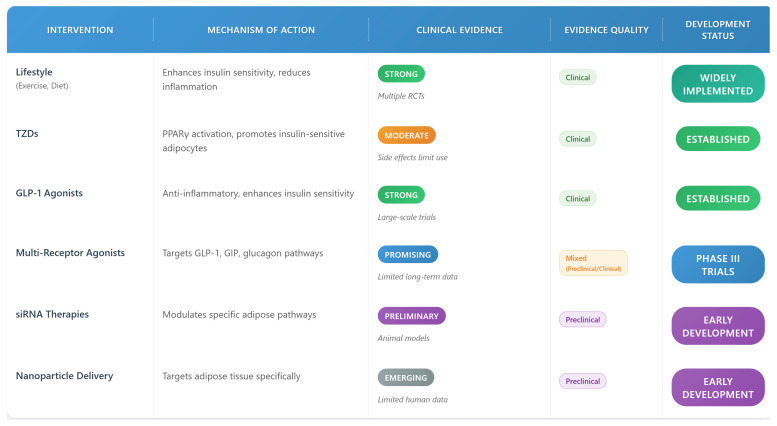
Current and emerging therapeutic approaches for adipose tissue insulin resistance: Comprehensive overview of therapeutic approaches for AT-IR, ranging from established lifestyle interventions to emerging targeted therapies. The figure summarizes mechanisms of action, clinical evidence strength, and current development status for each intervention category. Color-coded squares represent green for strong, orange for moderate, purple for preliminary, and grey for emerging, creating a visual “decoder” for readers.

The development of personalized therapeutic approaches based on individual patient characteristics and biomarker profiles represents an important future direction. Studies have begun to identify distinct patterns of adipose tissue dysfunction that may require different therapeutic strategies. This personalized medicine approach may ultimately lead to more effective treatments for metabolic disorders related to AT-IR. A comprehensive overview of current and emerging therapeutic approaches for AT-IR is provided in Figure 8, which summarizes mechanisms of action, clinical evidence strength, and development status for each intervention category.

### 8.4. Therapeutic Innovation and Future Directions

Tissue-specific drug delivery systems target adipose tissue to improve therapeutic efficacy and minimize systemic side effects. Nanoparticle-based approaches enable selective delivery to adipose tissue, addressing its unique metabolic properties [72]. Depot-specific strategies account for differences in visceral and subcutaneous fat, tailoring treatments to specific adipose tissue functions.

RNA-based therapeutics, such as siRNA and antisense oligonucleotides, modulate specific pathways in adipose tissue with high precision [73]. These approaches target disease-related mechanisms, offering potential for effective, low-risk treatments for AT-IR and related metabolic disorders.

## 9. Conclusions

This comprehensive review has examined the intricate relationship between adipose tissue insulin resistance and metabolic syndrome, revealing the central role of adipose tissue dysfunction in the pathogenesis of metabolic disease. The evidence presented demonstrates that adipose tissue insulin resistance represents not merely a consequence of metabolic syndrome, but rather a critical early event that initiates and perpetuates the cascade of metabolic derangements characteristic of this complex disorder. Adipose tissue heterogeneity, exemplified by Metabolically Healthy Obesity (MHO) and Metabolically Unhealthy Obesity (MUHO), ties to its capacity for healthy expansion. Patients with dysfunctional, visceral-heavy fat require distinct therapeutic strategies compared to MHO individuals. Personalized medicine for AT-IR must stratify patients not just by BMI, but by their adipose tissue phenotype—visceral versus subcutaneous—offering a tailored approach that unifies the review’s framework and provides a compelling direction for future research and clinical practice [23].

### 9.1. Key Findings and Clinical Implications

AT-IR drives MetS, acting as an early trigger for systemic metabolic dysfunction [2]. The discovery of leptin in 1994 revealed adipose tissue’s role as an endocrine organ [21], while studies by Kahn et al. (2003) showed that AT-IR alone can disrupt metabolic balance [23]. AT-IR often precedes systemic insulin resistance, offering a window for early intervention to prevent conditions like type 2 diabetes and cardiovascular disease [44]. Visceral fat is more inflammatory and insulin-resistant than subcutaneous fat, highlighting the need for targeted therapies [7,9,11]

### 9.2. Therapeutic Advances

Lifestyle interventions, such as exercise, improve insulin sensitivity, reduce inflammation, and enhance adipose tissue function by promoting mitochondrial activity and vascularization [59]. Pharmacological treatments like TZDs and GLP-1 receptor agonists enhance adipocyte function and reduce inflammation [60,61]. Emerging therapies, including multi-receptor agonists and adipose tissue remodeling strategies, target complex metabolic pathways [64,65]. Precision immune modulation, such as blocking CCL2/CCR2 or promoting M2 macrophage polarization, offers novel approaches to mitigate AT-IR-driven inflammation [64,65,70].

### 9.3. Future Directions and Challenges

Future research should integrate multi-omic approaches and single-cell genomics to uncover novel therapeutic targets in AT-IR [59]. Advanced animal models and human tissue studies are essential for clinical translation [8]. Clinical trials incorporating biomarkers and imaging will identify patients who benefit most from personalized therapies [58]. Specific research priorities for the next 5–10 years include: (1) elucidating the temporal sequence of AT-IR versus muscle insulin resistance, (2) identifying specific adipokine profiles driving disease progression, (3) developing cost-effective multi-omic platforms for clinical use, and (4) validating tissue-specific therapies in diverse human populations. However, barriers such as high costs of multi-omic technologies, limited accessibility in low-resource settings, and ethical concerns regarding data privacy in personalized medicine must be addressed to ensure equitable implementation [20]. Collaboration between researchers, clinicians, and policymakers will translate these advances into effective, accessible treatments, reducing the global burden of MetS [1].

### 9.4. Controversies and Knowledge Gaps

A key debate in metabolic syndrome research is whether adipose tissue insulin resistance (AT-IR) precedes muscle insulin resistance. Kahn et al. (2003) suggest AT-IR sparks early fat spillover into muscles and liver, causing broader insulin issues [23]. Others argue that muscle insulin resistance, driven by ectopic lipid buildup, starts systemic dysfunction. Animal studies often overstate AT-IR’s role due to genetic tweaks not matching human biology, while human data shows mixed results. Gaps remain in adipokine roles, like how they promote liver fat in NAFLD, disrupt hormones in PCOS, or fuel tumors in cancer. Cross-sectional human studies limit causal insights, and longitudinal data are needed to clarify timelines. Integrative research with human cohorts and omics tech is vital to resolve these issues.

## Abbreviations

The following abbreviations are used in this manuscript:

AT-IR	Adipose tissue insulin resistance
MetS	Metabolic syndrome
IRS	Insulin receptor substrate
PI3K	Phosphatidylinositol 3-kinase
TNF-α	Tumor necrosis factor-alpha
ER	Endoplasmic reticulum
UPR	Unfolded protein response
ISR	Integrated stress response
ROS	Reactive oxygen species
JNK	c-Jun N-terminal kinase
IKK-β	Inhibitor of nuclear factor kappa-B kinase subunit beta
ECM	Extracellular matrix
NAFLD	Non-alcoholic fatty liver disease
NASH	Non-alcoholic steatohepatitis
CVD	Cardiovascular disease
PCOS	Polycystic ovary syndrome
T2DM	Type 2 diabetes mellitus
FFA	Free fatty acids
TZDs	Thiazolidinediones
PPARγ	Peroxisome proliferator-activated receptor gamma
GLP-1	Glucagon-like peptide-1
GIP	Glucose-dependent insulinotropic polypeptide
